# Barriers and facilitators of child and guardian attendance in task-shifted mental health services in schools in western Kenya

**DOI:** 10.1017/gmh.2020.9

**Published:** 2020-06-30

**Authors:** Rosemary D. Meza, Sharon Kiche, Caroline Soi, Alya N. Khairuzzaman, Cristian J. Rivera Nales, Kathryn Whetten, Augustine I. Wasonga, Cyrilla Amanya, Shannon Dorsey

**Affiliations:** 1 Department of Psychology, University of Washington, Guthrie Hall 119A, Box 351525, Seattle, WA 98195, USA; 2 SMART Center, Psychiatry and Behavioral Sciences, University of Washington, 6200 NE 74th Street, Suite 110, Seattle, WA 98115, USA; 3 Department of Global Health, Harris Hydraulics Laboratory, University of Washington, 1510 San Juan Road, Seattle, WA 98195, USA; 4 Center for Health Policy and Inequalities Research, Duke Global Health Institute, Duke University, Box 90519, Durham, NC 27708, USA; 5 Terry Sanford Institute of Public Policy, Duke Global Health Institute, Duke University, Box 90239, Durham, NC 27708, USA; 6 Research Department, Ace Africa Kenya, P.O. Box 1185, 50200 Bungoma, Kenya

**Keywords:** Kenya, mental health, school, task-shifting, treatment attendance

## Abstract

**Background:**

Globally, nearly 140 million children have experienced the death of one or both parents, and as a result many experience higher rates of mental health problems. Trauma-focused cognitive behavioral therapy (TF-CBT) delivered by lay counselors has been shown to improve mental health outcomes for children experiencing traumatic grief due to parental loss; however, challenges with treatment attendance limit the public health impact of mental health services. This study used qualitative methods to assess barriers and facilitators of child and guardian attendance of school-based lay counselor delivered TF-CBT.

**Methods:**

Semi-structured interviews were conducted with 36 lay counselors (18 teachers; 18 community health volunteers) who delivered TF-CBT to explore their perceptions of barriers of facilitators of child and guardian attendance of group-based sessions delivered in schools.

**Results:**

Counselors identified attendance barriers and facilitators related to the delivery setting, resources, participant characteristics, intervention characteristics and counselor behaviors. The findings revealed a greater number of facilitators than barriers. Common facilitators included participant and counselor resources, counselor commitment behaviors and communication efforts to encourage attendance. Barriers were less frequently endorsed, with participant resources, child or guardian illness and communication challenges most commonly mentioned.

**Conclusions:**

Attention to barriers and facilitators of attendance in the context in which mental health interventions are delivered allows for identification of ways to improve attendance and treatment engagement and achieve the potential promise of providing accessible mental health services.

## Background

Globally, nearly 140 million children have experienced the death of one or both parents, with 52 million residing in African countries (UNICEF, 2015). Children orphaned in low- and middle-income countries (LMICs) are commonly exposed to multiple traumatic experiences, including child abuse and neglect, early parental death, communicable diseases, inadequate healthcare, poverty, and stigma (Kieling *et al*., 2011; Idele *et al*., 2018). Traumatic experiences are associated with long-lasting negative outcomes, including adverse effects on skill acquisition, decision making, and health outcomes (Monasch and Boerma, 2004; Andrews *et al*., 2006; Cluver and Gardner, 2007). Youth orphaned in African countries have higher rates of mental health problems including maladaptive grief, post-traumatic stress (PTS), anxiety, depression, suicidal thoughts, and behavior problems than youth who have not experienced parental death (Atwine *et al*., 2005; Cluver and Gardner, 2007).

Mental health services in LMICs are improving; nonetheless, resources remain extremely limited (Betancourt and Chambers, 2015). High population needs and low tax bases result in most LMIC governments allocating less than 1–2% of health budgets to mental health (Rathod *et al*., 2017). Most resources go to addressing seriously mentally ill adults (Thornicroft *et al*., 2010), while a median of merely 0.16% of children in LMICs with mental health disorders receive any treatment (World Health Organization, 2009). This treatment gap is exacerbated by shortage of human resources in LMICs, with many African LMICs experiencing significant mental health professional shortages (Bruckner *et al*., 2011). Funding and human resource limitations necessitate the identification of scalable and feasible strategies to address the treatment gap for children in LMICs (Eaton *et al*., 2011; Betancourt and Chambers, 2015).

Accumulating evidence suggests trauma-focused cognitive behavioral therapy (TF-CBT) – an evidence-based treatment for child trauma in the USA (Cohen *et al*., 2006) – is effective at reducing PTS, depression, anxiety and conduct disorder with trauma-exposed populations in LMICs, including those who experienced parental death (Dorsey *et al*., 2020b; Murray *et al*., 2015). To address limited human resources, efforts have utilized task-shifting, a process in which non-specialists with limited to no previous mental health experience provide treatment under supervision (World Health Organization, 2008). Studies utilizing a version of TF-CBT adapted to fit the local context demonstrate TF-CBT can be feasibly delivered using task-shifting and results in mental health symptoms improvements for youth in sub-Saharan Africa (Murray *et al*., 2013a, 2013b; O'Donnell *et al*., 2014). To enhance sustainability of these efforts, current research is examining the scale-up of task-shifted TF-CBT in schools (Dorsey *et al*., 2020a, 2020b).

The public health impact of efforts to increase access to mental health treatment can only be realized if services are utilized. When treatment dose is diminished by non-attendance, the positive clinical outcomes typically demonstrated in clinical trials are also diminished (Hansen *et al*., 2002). Caregiver treatment attendance is also important. In TF-CBT, caregivers enhance treatment outcomes through reinforcing therapeutic skills and psychoeducation (Dorsey *et al*., 2014). Enhancing attendance in group-based services can maximize potential returns on the investment of human and financial resources required for Evidence-Based Practice (EBP) delivery. When utilizing task-shifting in LMICs, non-attendance may lead to wasted human resources, as is the case in traditional outpatient services (Gordon *et al*., 2010).

Treatment attendance often suffers from community-based settings, with clients across diverse settings attending an average of three to five sessions – well below the recommended dose for most EBPs (Hansen *et al*., 2002). Caregivers in US-based publicly funded mental health settings attended an average of 42% of sessions (Wright *et al*., 2019). Though school-based services can reduce treatment access barriers and increase treatment enrollment, treatment attendance in schools remains suboptimal (Werner-Seidler *et al*., 2017). Less research on treatment attendance exists in LMICs; nonetheless, attendance challenges have been documented in LMICs. When examining perceptions of TF-CBT delivery in Zambia, poor attendance was the most common barrier reported (Murray *et al*., 2014).

Attendance barriers have been identified, including client and treatment characteristics, resource requirements, treatment setting, and culture, though these mainly stem from research in high-income country (HIC) settings. Caregiver disappointment with mental health services, distrust of the system (Contractor *et al*., 2012; Baker-Ericzén *et al*., 2013) and negative attitudes toward mental health service use (Logan and King, 2001) are associated with decreased service utilization. While school-based services can reduce cost and transportation barriers (Weist *et al*., 2017), they can also engender other barriers. Pella and *et al*. (2018) studied children's perceived barriers to attending school-based treatment in the USA and found the most common barriers were concerns about missing classroom work and stigma about receiving mental health services. In LMICs, resource barriers including cost, transportation challenges, and the amount of time and opportunity cost from time spent attending sessions interfered with treatment engagement (Patel *et al*., 2011; Murray *et al*., 2014). Low intervention acceptability and stigma about accessing care have also been barriers to treatment use in LMICs (Patel *et al*., 2011).

### Current study

The parent study, Building and Sustaining Interventions for Children (BASIC), uniquely examines conditions that create an enabling context to scale-up and sustain TF-CBT in Kenya. Building on that goal, we used qualitative methods to examine barriers and facilitators of child and guardian attendance of lay-counselor delivered TF-CBT to inform our ultimate goal of facilitating a treatment delivery context that supports treatment attendance. Lay counselor perspectives were prioritized due to their familiarity with the treatment delivery context and counselor and consumer behaviors that may have helped or hindered attendance.

## Methods

The parent study, BASIC, is a NIMH-funded study of TF-CBT delivered by teachers in the education sector and community health volunteers (CHVs) in the health sector in Western Kenya (see Dorsey *et al*., 2020a, 2020b). BASIC is a stepped wedge cluster randomized trial that includes seven sequences. Forty primary schools and their surrounding 40 village clusters, referred to as communities, were randomly selected from the 137 primary schools in Kanduyi Constituency and randomly ordered into seven sequences. Sequences initiate delivery of TF-CBT sequentially. Sequence 1, in this study, has ten schools and 10 communities. Data from sequence 1 is used to inform implementation in sequences 2–7.

### Intervention

Counselors delivered an adapted version of TF-CBT, subsequently referred to as Pamoja Tunaweza (PT), for single- or double-orphaned youth and their guardians. PT is delivered by lay counselors and involves eight group-based sessions and two-to-three individual sessions mid-group for imaginal exposure.

### Procedures

#### Recruitment and consent

Youth aged 11–14 were eligible to receive PT if one or both of their parents had died and they (1) were aware the parent(s) had died, (2) were attending a study-enrolled school, (3) lived at home with at least one adult (*v.* group home or congregate care setting), and (4) were experiencing PTS and/or prolonged grief. Schools and the local health facility via the Community Health Extension Workers (CHEWs) and CHVs provided the Kenyan research interviewers from Ace Africa with lists of orphaned youth in each village cluster. Guardians of orphaned youth were contacted over a phone, or in-person if a phone number was not available, to describe the study and schedule an in-person appointment. If guardians expressed interest, a pair of Ace Africa interviewers visited the family at their home to describe the study aims and participant involvement. Written informed consent was collected from guardians for themselves and the youth, and upon guardian consent, written assent was collected from youth. If both agreed to participate, youth and guardians completed the Child PTSD Symptom Scale (CPSS) and youth reported on prolonged grief using the Inventory for Complicated Grief (ICG) to determine youth clinical eligibility. Youth were clinically eligible if they scored ⩾18 on the CPSS by youth or guardian report or 35 on the ICG based on youth report. Both measures have demonstrated acceptable reliability among Kenyan youth who experienced parental death (Dorsey *et al*., 2020b). The youth and guardian measures were administered simultaneously – one interviewer with the child, one interviewer with the guardian – to reduce the time burden on the family. Interviews typically lasted about 40 min. Guardians received an incentive of 500 Kenyan Shillings (KSh) and youth received 100 KSh. For the initial group, guardians of clinically eligible youth randomly drew an envelope determining their randomization to receive PT in CHV or teacher-led groups. In subsequent groups, after 16 clinically eligible youth were identified (or the maximum available up to 16), study staff used a computer-based randomization tool to assign youth to CHV or teacher groups; guardians were informed of their study condition prior to the start of groups. Of the 280 families who were eligible in sequence 1, no guardians or youth declined participation.

Following an initial BASIC sensitization meeting with each site, Ace Africa worked with site leadership to recruit three lay counselors. Site leaders nominated teachers and CHVs to participate in BASIC who were good with children, may have had counseling experience, had time to deliver PT, and had no immediate plans to leave their school/area. Interviewers met with nominated teachers and CHVs to describe the study aims and to obtain written informed consent.

#### Intervention training and delivery

The CHV and teacher counselors were trained to deliver PT in January 2018 and delivered two sequential PT groups in schools during two academic terms. The education sector delivered PT in schools and opted to conduct PT during games time, a 1-h period where students participate in clubs and sports. The health sector had autonomy to decide the location of PT delivery given CHVs' services are integrated within the community. During sensitization meetings in schools, Head Teachers welcomed CHVs to deliver PT in schools given the availability of space. To date, all health facilities have opted to deliver PT in schools.

#### Data collection

Following delivery of two PT groups, qualitative interviews were conducted with a sample of CHV and teacher counselors from twelve sites, six sites per sector, in sequence 1. Sites were purposively sampled to maximize variation in characteristics believed to potentially impact PT delivery. Schools were sampled based on urban and rural classification, supervisors' subjective rating of Head Teacher leadership, number of teachers, student–teacher ratio, and the supervisor for that site. Health facilities were sampled based on urban and rural classification, religious make-up, population size of the village cluster served by CHVs, whether CHV counselors had responsibilities in addition to PT (e.g. involved in sanitation program), sources of CHV funding, and the supervisor for that site. Data were collected between July and August 2018. Teacher counselor interviews were conducted in English, whereas CHV counselor interviews were conducted in Swahili to accommodate language preferences. Procedures were approved by the Institutional Review Boards at Duke University, Kenya Medical Research Institute, and National Commission for Science Technology and Innovation. Counselors received an incentive of 500 KSh, approximately $5, for interview participation and weekly for phone credits to communicate with PT participants. On average, guardians received 200 KSh and children received 50–100 KSh for transportation weekly, depending on weather conditions and distance traveled to school for sessions.

### Participants

Participants were 36 lay counselors (18 teachers; 18 CHVs) from six sites per sector. Three lay counselors from each sampled site (two child group counselors; one guardian group counselor) who delivered PT in sequence 1 were interviewed.

### Qualitative interviews

Data were collected using a semi-structured interview guide including open-ended questions about counselors' experience delivering PT and specific questions regarding barriers and facilitators of PT delivery related to resources, rewards and incentives, supervision, leadership, workload, and roles within the delivery context. The guide was developed in English and translated to Swahili. Each interview was conducted by an interviewer and notetaker from Ace Africa with previous data collection and qualitative interviewing experience. The interviewer and notetaker took detailed notes of responses during interviews. Notes were reviewed and consolidated to ensure consensus. All interviews were audio-recorded. During initial interviews, the study team reviewed audio recordings and notes immediately post-interview to ensure consistency and provide feedback for interviewers and notetakers to improve interviewing process and notetaking.

### Data analysis

Thematic analysis was conducted by initially taking a deductive approach that considered pre-identified multilevel facilitators and barriers that might impact child and guardian treatment attendance by a team of five coders. These included (1) school characteristics, (2) counselor behaviors, (3) youth and guardian characteristics, (4) resources required for treatment participation, and (5) intervention characteristics. Three authors (RM, SK, and CS) initially applied the existing coding framework to a subset of English interviews and emergent codes were compared, refined, and organized into thematic categories iteratively throughout analysis. Senior authors (RM, SK, and CS) reviewed themes and sub-themes that emerged from the final coded data. The updated codebook was applied to 18 Swahili (SK and CS) and 18 English (RM, AN, and CR) interview notes. Interviews were independently coded by two coders and discrepancies were resolved through consensus discussions. If consensus was not reached, a third coder was consulted. The first author (RM) participated in all consensus discussions with Swahili coders to ensure consistency across languages. We calculated frequencies and percentages of codes. Quotes in Swahili were translated into English and marked with an asterisk (*).

## Results

Nearly all teacher and CHV counselors reported barriers (93%) and facilitators (98%) of attendance (see Tables 1 and 2). Teachers reported an average of 3.7 (s.d. = 2.2) barriers while CHVs reported an average of 2.0 (s.d. = 1.9) barriers. The average number of facilitators reported was 5.1 (s.d. = 2.1) and 3.9 (s.d. = 2.5) for teachers and CHVs, respectively.

**Table 1.** Barriers of youth and guardian attendance (teacher: *N* = 18; CHV: *N* = 18)

Attendance barriers	Teacher, *N* (%)	CHV, *N* (%)
Delivery setting		
*Structure and procedures*		
Preparation and sitting for primary school national graduation exam	4 (22)	3 (17)
Students with unpaid non-tuition fees sent home	2 (11)	2 (11)
Student absent from school	1 (6)	3 (17)
School and public holidays	2 (11)	1 (6)
Class/school schedule not adjusted	1 (6)	1 (6)
*Staff behaviors*		
Staff not releasing students from classes	4 (22)	1 (6)
Resources		
*Participant resources*		
Heavy rain increasing transportation fees and blocking roadways	8 (44)	2 (11)
Insufficient transportation stipend	1 (6)	0 (0)
Guardian expectation of financial compensation for participation	1 (6)	0 (0)
*Counselor resources*		
Insufficient phone credits or transportation stipend to contact guardians about attendance or deliver make-up sessions	2 (11)	1 (6)
Participant characteristics		
*Buy-in/engagement*		
Guardian and/or youth unwillingness to attend	4 (22)	4 (22)
*Competing demands*		
Youth or guardian conflict (e.g. funeral, appointment)	5 (28)	4 (22)
Guardian work conflict	4 (22)	4 (22)
Farming responsibilities or work responsibilities at home	2 (11)	0 (0)
*Physical and mental health*		
Youth and guardian illness	8 (44)	2 (11)
Guardian alcohol use	2 (11)	0 (0)
Psychological symptom severity (e.g. trauma triggers)	1 (6)	1 (6)
Intervention characteristics		
Proximity of PT delivery to participants' home and work	3 (17)	0 (0)
Requirements for same guardian attendance	1 (6)	1 (6)
Counselor behaviors		
*Communication*		
Communication challenges	3 (17)	7 (39)
Miscommunication about or changes to the session schedule	3 (17)	1 (6)

**Table 2.** Facilitators of youth and guardian attendance (teacher: *N* = 18; CHV: *N* = 18)

Attendance facilitators	Teacher, *N* (%)	CHV, *N* (%)
Delivery setting		
*Staff behaviors*		
Staff releasing student from class	11 (61)	1 (6)
Staff support behaviors	0 (0)	7 (39)
*Leader support*		
Leader support behaviors	4 (22)	7 (39)
Participants allowed to attend PT session even if school fees were unpaid	1 (6)	1 (6)
Resources		
*Participant resources*		
Transport stipend provided to youth or guardian	10 (56)	8 (44)
Sending public transportation to pick up a youth or guardian for session	2 (11)	1 (6)
*Counselor resources*		
Resources to call or visit guardians or youth	10 (56)	6 (33)
Use of personal resources to call, visit, or make youth and guardian attendance possible	4 (22)	5 (28)
Participant characteristics		
*Buy-in/engagement*		
Realizing the non-monetary benefits of participating in PT	0 (0)	1 (6)
*Physical and mental health*		
Spoke to or warned guardians who arrived under the influence of alcohol	2 (11)	0 (0)
Quizzed absent guardians absent to encourage attendance	1 (6)	0 (0)
Intervention characteristics		
Supervisor support	3 (17)	2 (11)
PT delivered during school day	2 (11)	0 (0)
PT delivered nears participants homes	0 (0)	1 (6)
Counselor behaviors		
*Counselor commitment/behaviors*		
Conducting make-up sessions	9 (50)	7 (39)
Adjusting the time of PT activities	11 (61)	4 (22)
Extra effort to deliver PT to youth and guardians	1 (6)	7 (39)
*Communication*		
Speaking to participants to encourage attendance	16 (89)	9 (50)
Informing participants about session schedule changes	1 (6)	1 (6)
Informing participants of program expectations in advance	1 (6)	1 (6)
Providing guardians with counselor phone number	1 (6)	0 (0)

### Delivery setting

#### Structure and procedures

School structures and procedures were identified as attendance barriers, though they occurred infrequently. The most frequently endorsed procedural barrier was the primary school national graduation exams. Grade (class) 8 students sit for exams at the end of the school year to advance to secondary school. These students were busy throughout the year with exam preparation activities which were prioritized over other extracurricular activities, including PT.

One counselor explained, ‘Those candidates… you find that sometimes you need them, but they are doing an exam… In most cases, the problem came from these candidates. Yeah, so you might need them and find that maybe they are worn out. Like the last two weeks, class 8 were to go to [school]on the day you were supposed to have a session, they were to go there so that they could release… the results of the exams… so you find that some of these pupils miss the session. But we organized and make them up on the following day to give them what the others had done’. (Teacher Counselor)

Though Kenyan government-funded primary schools do not require tuition, non-tuition fees are required to support school activities. Schools often send students with unpaid fees home until fees are paid. When students from PT groups were sent home, counselors often were unaware until groups began and had to hire a motorbike to bring students back in time for session. Similarly, if students were absent from school for other reasons, their absence precluded them from attending session. Other infrequent context-based barriers to attendance included not adjusting the school or class schedule when school activities interfered with a PT session and holidays coinciding with PT session resulting in school closure.

Describing the impact of youth being sent home for unpaid fees, one counselor stated, ‘And then sometimes some children have been sent home due to [non-tuition fees]… if the child has been sent home [he/she] will not return back to school and when you go for PT sessions you don't find them and I was forced to go and look for the child at their home and bring them back for PT session’. (CHV counselor)*

#### Staff behaviors

Staff releasing students from classes or school activities to attend PT was among the most frequently endorsed attendance facilitators by teacher counselors. Students were often in class when sessions began, requiring the cooperation of teachers to release them. Occasionally, teacher counselors also identified students not being released from class as a barrier. They often addressed this barrier by gaining the Head Teacher's support for releasing students, prompting teachers to comply. Among CHVs, wide-ranging staff support was identified as facilitating attendance, including teachers locating PT participants and ensuring timely attendance. A variety of staff, including administration, teachers and gatekeepers, collaborated to ensure students and guardians were able to attend sessions.

#### Leader support

Support from leadership was characterized as facilitating attendance, especially among CHV counselors. This involved the Head or Deputy Head Teacher ensuring students were in session and sending other students to find youth not in session. When CHVs conducted individual visits in youth's homes, the Village Elder provided assistance in gaining access to their homes to conduct sessions. School leaders also occasionally adjusted school policies to allow students with unpaid fees to remain at school to attend session.

#### Resources

Many barriers and facilitators were identified related to resources required to attend session, motivate attendance, or for counselors to travel or speak to participants who missed sessions.

*PT participant resources*: The transportation stipend for guardians and youth was commonly noted as facilitating attendance. However, one counselor noted the stipend was insufficient and others reported hiring a motorbike to pick up youth or guardians unable to afford transportation. During the rainy season, heavy rain significantly increased transportation fees and made roadways to schools impassable, posing a barrier to guardian attendance.

*Counselor resources*:

Counselors reported resources facilitated their ability to support participant attendance. These primarily included phone credits to call and remind guardians about sessions or schedule make-up sessions with absent participants. On occasion, counselors were able to borrow a phone or received transportation funds to conduct make-up sessions. Though uncommon, some counselors reported phone credits or transportation funds were insufficient. In a demonstration of their commitment to delivering PT to participants, some counselors used their own money to support attendance. This was primarily used for transportation funds to reach participants. One teacher counselor reported they and other staff used their own money to cover medical expenses for a youth who was absent due to illness.

The counselor noted, ‘One of the children was… not feeling well … The [youth] was supposed to attend the session … So, the [youth] was sick but one of the staff members, in fact, they contributed some amount. They said, ‘we don't want [youth] to miss, because we've been seeing this child since last year. [The youth] was so traumatized with the loss of the parents because [the youth] lost both parents.’ So, we talked about it, we gave out some amount, the [youth] was taken to the hospital, and the next day [the youth] came back’. (Teacher Counselor)

#### Participant characteristics

*Buy-in/engagement*. PT participants' buy-in and engagement, competing demands, and mental and physical health emerged as barriers and facilitators of attendance. Some guardians and, to a lesser extent, youth expressed an unwillingness to attend due to a lack of interest or understanding of the value of PT.

Describing guardians' unwillingness to attend, a counselor stated, ‘And then on the side of the guardians, it was also a bit challenging because some of them would not come, they would have a lot of excuses. So, you keep on calling them, calling them, you call a guardian like four times. [A guardian is] telling you ‘I'm on the way coming.’ And then, maybe [they don't] show up’. (Teacher Counselor)

One CHV counselor noted attendance improvements when youth and guardians recognized the non-monetary benefits of participating in PT, including improvements to mental health.

*Competing demands.* Competing demands were a barrier, especially for guardians. Several counselors reported guardians had conflicts including funerals, community events, and appointments. Since groups occurred during business hours, some guardians' work schedules conflicted with attendance. Though rare, some youth missed sessions due to farming or housework responsibilities.

*Physical and Mental Health.* Participants' poor physical and mental health were noted as barriers to attendance. PT participant illness was noted as a barrier, especially by teacher counselors. Less commonly, guardian alcohol use interfered with attendance. To address this, counselors reported speaking with and warning guardians who arrived intoxicated. One counselor quizzed guardians on content from missed sessions to encourage attendance. In rare cases, a participant's symptom severity impacted their ability to attend session.

#### Intervention characteristics

Details related to the intervention location, timing, participant guidelines and supervision emerged as barriers and facilitators. Holding PT sessions during the school day was identified as a facilitator due to the availability of youth. However, in some regions – especially rural areas – some families did not live near school. This distance posed a barrier to attendance, especially for guardians who traveled to school for sessions.

When enrolling in groups, a single guardian was asked to participate in all sessions to ensure guardians learned the full intervention content and create a cohesive guardian group. Counselors reported not allowing guardians to share the role of attending groups occasionally obstructed consistent attendance. Support received from PT supervisors was occasionally identified as facilitating participant attendance. This primarily involved reminding counselors to call and remind guardians about upcoming sessions and helping counselors to solve problems related to participant attendance.

#### Counselor behaviors

*Counselor commitment.* Counselors reported engaging in various behaviors to facilitate attendance or mitigate the effect of non-attendance. Over half of counselors reported conducting make-up sessions with families to cover missed content. This involved calling guardians to coordinate a separate time to deliver content in schools, at home or in a location convenient to the participant, such as the marketplace. Make-up sessions emerged as an important method for participants to receive the full intervention, but also increased burden on counselors. In some cases, counselors temporarily delivered two sessions per week to accommodate class 8 youth who were unable to attend sessions during exam preparation. Counselors also adjusted PT session times to facilitate participant attendance. Examples include, delaying session until all guardians arrived and rescheduling or extending sessions to allow guardians enough time to share. Adjustments could result in significant delays to sessions starting, requiring a greater time commitment from counselors and participants.

Counselors often exceeded expectations to ensure participants received PT. These extra effort behaviors were more common among CHVs, who had greater flexibility and time, including repeatedly visiting homes to find participants for make-up sessions and individual visits, staying long past scheduled session times to ensure participants received the full content, waiting hours until exams ended to deliver PT, and repeatedly delivering in-home sessions for sick guardians.

Describing efforts to conduct an individual visit, one counselor stated, ‘I had a challenge during individual visits. Sometimes you don't find the child at home and when you ask the guardian, she tells you ‘oh the child went to such and such a place.’ I am then forced to take a bicycle or motor bike to go and look for that child. I can arrive there and they tell me ‘oh the child has gone back home’ so I am looking for the child until the evening and you don't find her and you have to go back the next day because you want to speak with that child… and since you are committed you will look for the child until you talk to her’. (CHV counselor)*

*Communication.* Efforts to communicate with and encourage participants to attend sessions were the most common facilitators of attendance. Counselors repeatedly encouraged attendance and reminded participants. They also recruited others to help with encouraging attendance, including Head Teachers, supervisors, school staff and other youth and guardians.

Describing collaborative efforts to encourage guardian attendance, one counselor stated, ‘We had to involve the supervisor. She went into [their] house. They sat, she explained [the] importance of [the guardian] attending the meetings and try[ing] to come early. So, to some extent [they] adjusted… [The supervisor] went three times… and other times [the supervisor] would call [them] personally on the phone… There's a staff mate of ours who is [their] neighbor. So, we also told her to tell [them]… to try to be coming for the meetings and try to be coming early. So, through those – all those efforts is when we realized [they] changed a bit, but not like fully like those [other guardians]. But we pushed on until we completed the session with perseverance’. (Teacher Counselor)

To facilitate attendance, counselors clarified program expectations and informed families about session changes. However, communication challenges were a common barrier, including difficulty reaching guardians by phone or in-person and occasionally lacking phone credits to remind guardians. Counselors reported some miscommunication about session schedules that negatively impacted attendance.

## Discussion

This study examined barriers and facilitators of attendance in a task-shifted intervention delivered in schools to inform development of strategies future implementing sites could use to improve attendance. Task-shifting and school-based delivery hold promise for increasing youths' access to mental health care (Fazel *et al*., 2014), but benefits of increased access can only be realized if participants attend and engage in care.

Schools are recommended settings for scaling up of youth mental health services in LMICs (Patel *et al*., 2013a, 2013b); however, some features of school-based delivery can create challenges for attendance if unaddressed. In our study, the greatest number of unique barriers were related to school structure and procedures. Competing education priorities in schools occasionally interfered with attendance, with preparation for national primary school graduation exams noted most frequently. Striking a balance between education priorities and treatment attendance will be necessary in schools where education is the primary priority and competing initiatives often arise (Owens *et al*., 2014). Some academic activities may have to take priority. For instance, counselors opted to exclude graduating grade 8 students from future PT groups because schedule conflicts were unresolvable. Other structural and procedural barriers may be resolved by gaining support from teachers and school leadership, as their support has been essential in incorporating mental health interventions in schools in the literature (Forman and Barakat, 2011) and in our study (e.g. releasing students to attend).

Financial resources provided to counselors and participants were among the most commonly endorsed facilitators. These included financial resources for counselors to schedule and provide make-up sessions and individual visits as well as transportation funds for participants. While the parent study intentionally kept study-provided financial resources to a minimum for sustainability (Rathod *et al*., 2017), results suggest some financial resources are important. Previous studies have found transportation costs to be a barrier to attending sessions in low-resource settings (Murray *et al*., 2014; Patel *et al*., 2011) and without some transportation assistance for guardians, attendance may suffer. Similarly, the increased cost of transport during heavy rains was a barrier to attendance. A cost analysis conducted as part of the parent study (Dorsey *et al*., 2020a, 2020b) will calculate all costs associated with PT delivery, which may aid policymakers as they consider integrating services into their budget. Though funding transportation may not be a funding priority, the return on investment will be greater for governments if participants can attend services.

The importance of financial resources to support attendance highlights a tension in scale-up efforts. EBT scale-up has been limited, in part, by a lack of funding (Eaton *et al*., 2011). Minimizing implementation costs is essential, but mental health trials in LMICs have often required some financial support. For instance, funds have been provided for communication between counselors and their supervisors when clinical emergencies arose and to contact participants for follow-up (e.g. Murray *et al*., 2013b; Weiss *et al*., 2015). Without such support, ethical challenges arise regarding expecting lay counselors to deliver services as they may face trade-offs between using their own money or neglecting certain delivery activities such as contacting supervisors for support. The financial resources provided for counselors and families – though limited – may hinder the sustainability of PT delivery and family attendance. In the parent study, this tension is addressed by continuously aiming to reduce costs where possible and also measuring all costs to inform efforts to attain locally sustainable funding. To this end, subsequent sequences no longer provided incentive payments for child and guardian attendees. Anecdotally, supervisors report this reduced guardian, but not child attendance. Building on Kenya's recent National Mental Health Policy (Republic of Kenya, 2014), the parent study aims to use these cost calculations to aid in acquiring sustainable funding.

Findings revealed CHVs played a unique role in supporting attendance and demonstrated the importance of collaboration across sectors. CHVs are a bridge between the community and health facilities (Ayala *et al*., 2010), bringing services directly to the community. CHVs described engaging in behaviors that exceeded counselor expectations to bring PT to participants. This involved meeting participants at home or in the marketplace to ensure those who missed sessions received the content. Despite the increased time and effort this required, CHVs reported PT delivery was highly feasible (Dorsey *et al*., 2019). It is possible that CHVs' extra-effort behaviors have been motivated, in part, by the external oversight from study-imposed structures such as tracking and supervision – potentially limiting their sustainability. To support the sustainability of PT and the investment of the individuals who have made it possible, the project has fostered local ownership of PT among the schools and communities where it is being implemented. To that end, local stakeholders, including a subset of counselors from sequence 1, guided the development of strategies to support PT implementation, including attendance. Teacher and CHV counselors from sequence 1 function as volunteer coaches for sites that implement PT after them, to provide guidance and support the use of sustainable methods to deliver PT and encourage attendance. Support CHVs received from school staff and leadership also facilitated attendance. Partnerships across healthcare platforms or between health, academic and non-governmental organizations have improved mental health treatment access (Acharya *et al*., 2017), but fewer examples of health and education collaboration exist. Collaborative efforts between both sectors may be a promising method to increase access by delivering treatment in an accessible setting and using CHVs to bridge treatment gaps through their community engagement role.

Few barriers related to intervention characteristics were identified, and those that emerged were not related to intervention content, which may reflect attention to PT's acceptability (O'Donnell *et al*., 2014). This is promising as the acceptability of western-based interventions has been a barrier to treatment engagement in LMICs (Patel *et al*., 2011). Similarly, although stigma associated with accessing care has been a barrier to attendance in LMICs (Patel *et al*., 2011) and school-based HIC settings (Pella *et al*., 2018), stigma did not emerge as a barrier in our study. Delivery by CHVs has been shown to reduce stigma and increase service engagement (Balaji *et al*., 2012). Features of the delivery model, including school-based delivery by familiar teachers and CHVs may have reduced stigma in the current study.

Study findings were used to assist stakeholders in selecting strategies to improve attendance in subsequent sites implementing PT, with emphasis on addressing modifiable barriers without using additional resources. Stakeholders engaged in a method for developing tailored strategy plans (Lewis *et al*., 2018) that were used to coach future sites in supporting attendance and PT implementation (Dorsey *et al*., 2020a, 2020b). Some aimed to *directly* impact attendance, such as developing procedures for regular attendance reminders and using the first session to thoroughly sensitize participants about PT benefits and expectations to motivate attendance. Others intended to *indirectly* target attendance. Leadership support has aided in the integration of interventions in schools (Forman and Barakat, 2011), therefore one *indirect* strategy involved gaining Head Teacher buy-in to resolve schools' structural and procedural barriers to attendance by inviting them to PT training.

### Strengths and limitations

Study strengths include the in-depth examination of treatment attendance barriers and facilitators in LMICs. The realistic study conditions, including lay counselor delivery in school-based settings is a particular strength. Purposive sampling prioritized data collection across diverse sites. However, additional themes may have emerged from unsampled sites. Due to the parent study's focus on implementation, our study includes only counselor perspectives on barriers and facilitators of attendance. The inclusion of youth and guardian perspectives would have likely enriched the study findings and not including their perspective may have resulted in missed themes. Finally, we were unable to link themes to attendance rates.

## Conclusions

The findings revealed a predominance of facilitators of child and guardian attendance in lay counselor delivered PT in Kenyan schools. Common facilitators included resources, counselor commitment behaviors and communication efforts to encourage attendance. Barriers were less frequently endorsed, with participant resources, illness and communication challenges most commonly mentioned. Attention to barriers and facilitators of attendance in the context in which mental health interventions are delivered allows for identification of ways to improve attendance and treatment engagement and achieve the potential promise of providing accessible mental health services.
